# Editorial for Special Issue: Gel-Based Drug Delivery Systems for Cancer Treatment

**DOI:** 10.3390/gels10110680

**Published:** 2024-10-23

**Authors:** Haoan Wu, Xingchun Gao

**Affiliations:** 1 Yale School of Medicine, Yale University, New Haven, CT 06520, USA; gxc199281003@163.com; 2 School of Biological Science and Medical Engineering, Southeast University, Nanjing 210096, China

## 1. Introduction

In recent years, hydrogel-based cancer drug delivery systems have developed rapidly due to the versatility of hydrogels. From the perspective of hydrogel design, injectability, temperature, and pH sensitivity increase the possible applications of hydrogels. The objects of hydrogel delivery comprise large- and small-molecule drugs, nanoparticles and vaccines. Hydrogel therapy also involves tumor types ranging from bone cancer to skin cancer. Therefore, hydrogels have become an important area of research in cancer treatment.

**Figure 1:**
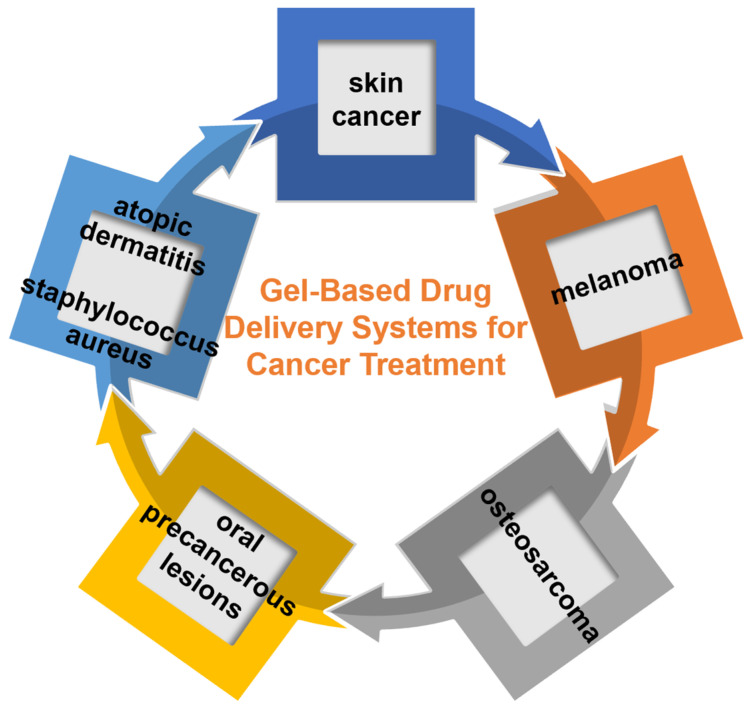
Summary of gel-based drug delivery systems for cancer treatment presented in this Special Issue of *Gels*.

This Special Issue brings together contributions from leading scholars in the field, with publications from 60 researchers representing more than 10 different countries worldwide. These countries include Bulgaria, Canada, India, Italy, Portugal, Saudi Arabia, Serbia, South Africa, Spain, and the USA. Importantly, these innovative areas of research include essential formulations and optimization processes for hydrogels, along with specific applications covering osteosarcoma, skin cancer, atopic dermatitis, oral precancerous lesions, melanoma, and staphylococcus aureus, among others (Figure 1).

## 2. Contributions

Hydrogels have been employed to avoid the side effects of chemotherapeutic drugs, improving the accumulation and maintenance of drug release at the tumor site. Tanga et al. discusses the challenges in designing thermoresponsive hydrogels for cancer treatment and offers suggestions to overcome these challenges. Additionally, the shortcomings in the administration process are also examined [1]. In their review of hydrogel applications, Tharakan et al. reviewed the use of novel stimuli-responsive hydrogels in osteosarcoma treatment, which facilitate the controlled release of anti-tumor drugs and modulate their biomechanical properties [2]. Slavkova et al. reviewed the most commonly used techniques for topical drug delivery via hydrogel-based topical formulations in skin cancer treatment. They also discussed the combinatorial formulation of nanocarrier-loaded hydrogels, focusing on ways to improve drug delivery [3].

In their research paper, Alam et al. designed and optimized a formulation of strychnine-loaded transliposomes (STCN–TLs) for the dermal administration of STCN to treat skin cancer. Their STCN–TL formulation demonstrated a 2.5-fold increase in permeability compared to the STCN solution and might be a suitable nanocarrier for the cutaneous distribution of STCN in the treatment of skin cancer [4]. Focusing on another skin disease, Kharwade et al. constructed tioconazole-loaded novel transferosome carriers (TTFs) for the treatment of atopic dermatitis. They demonstrated that a low dose of F2-TTFsH is a promising tool that effectively targeted the skin for the topical delivery of tioconazole to treat atopic dermatitis symptoms [5].

As one of the most aggressive forms of skin cancer, the treatment of melanoma is hindered due to the limited effectiveness of current therapies and the toxicity of conventional drugs. Trombino et al. created microparticles based on silk fibroin and silk-derived proteins loaded with idebenone to treat melanoma. They found that idebenone-loaded silk protein microparticles could effectively reduce the proliferation of melanoma cells without cytotoxicity. The designability of the hydrogel makes it versatile [6]. Pierfelice et al. combined photodynamic therapy (PDT) with hydrogel to investigate the effects of this treatment on the premalignant oral mucosa cell line. They found that the combination therapy exerted inhibitory effects on the proliferation and migration of DOK cells by inducing ROS and necrosis [7]. Hydrogels are widely used in studies on a variety of diseases, including in antimicrobial research. Alnasser et al. established an isoeugenol-loaded liposomal carrier gel to develop an antimicrobial therapy. The developed isoeugenol-liposomal gel was safe, with 75.95 ± 3.79% drug release after 24 h. The minimum inhibitory concentration (MIC) was 8.236 µg/mL. They predicted that adding isoeugenol to the liposomal gel could provide a potential means of methicillin-resistant Staphylococcus aureus treatment [8].

A detailed study was also conducted of the composition and properties of the hydrogel preparation. Matos et al. investigated the topical formulations of *Acanthus mollis* L. leaf and the optimization of their release of active compounds and their topical bioavailability. The results showed that bioactive compound DIBOA exhibited the release kinetics of the Weibull model for the Carbopol and Pluronic F-127 formulation, suggesting that it can be used in inflammation treatment and tyrosinase inhibition [9]. Penev et al. investigated the possibility of reformulating ClearView products in order to minimize the dose rate effect by optimizing the tetrazolium salt and gellan gum concentrations. The results showed excellent geometric and dosimetric registration, with a gamma passing rate (at 10% minimum dose threshold) of 99.3% for dose differences and a distance from the agreement criteria of 3%/2 mm. The new formulation may allow for quality assurance in complex treatment plans, which is important to their practical clinical application [10].

## 3. Conclusions

We look forward to providing readers with insights into hydrogel-based cancer therapy in this Special Issue. There is no doubt that the development of this emerging field has attracted contributions from different disciplines, such as biology, chemistry, physics, and materials. We also hope that readers will find the various topics presented here interesting and inspired, and that the studies will lead to the promotion of a wide range of commercial applications for hydrogels in a variety of different fields.

Given the rapid development of the field, as guest editors, we realize that presenting all these topics in one issue is limited. However, the interdisciplinary and high-quality nature of these articles leads us to believe that we can provide readers with an up-to-date and broad panorama of the use of these gels in the field of cancer therapy.
